# Next-generation metabolomics in lung cancer diagnosis, treatment and precision medicine: mini review

**DOI:** 10.18632/oncotarget.22404

**Published:** 2017-11-11

**Authors:** Li Yu, Kefeng Li, Xiaoye Zhang

**Affiliations:** ^1^ Department of Oncology, Shengjing Hospital, China Medical University, Shenyang, Liaoning, China; ^2^ School of Medicine, University of California San Diego, San Diego, CA, USA

**Keywords:** next-generation metabolomics, lung cancer, precision medicine, biomarker, pathogenesis

## Abstract

Lung cancer is the leading cause of cancer-related death. Next-generation metabolomics is becoming a powerful emerging technology for studying the systems biology and chemistry of health and disease. This mini review summarized the main platforms of next-generation metabolomics and its main applications in lung cancer including early diagnosis, pathogenesis, classifications and precision medicine. The period covers between 2009 and August, 2017. The major issues and future directions of metabolomics in lung cancer research and clinical applications were also discussed.

## INTRODUCTION

Lung cancer is a kind of malignant tumor that starts in the bronchus and lung. According to the morphology of cancer cells, lung cancers are generally divided into two categories: non-small cell lung cancer (NSCLC) and small cell lung cancer (SCLC), and the ratio of NSCLC to SCLC is about 4:1. With the continuous improvement in medical level, the mortality rate of lung cancer is decreasing; however, currently lung cancer is still the cancer with the highest mortality rate, and the 5-year postoperative survival rate in lung cancer patients is only around 13% [1]. The main reasons include: (1) The early clinical symptoms of lung cancer is not obvious and effective early diagnosis approach is lacked; (2) The pathogenesis and molecular subtyping of lung cancer is relatively complex; (3) The individual differences are significant and therapeutic precision medicine regimens that are specific to different types of lung cancers and individual patients are absent. Therefore, currently the main target of lung cancer research is to search new early diagnosis technology and markers, further explore the molecular mechanisms of lung cancer pathogenesis and scientifically and effectively evaluate the therapeutic effect.

**Figure 1:**
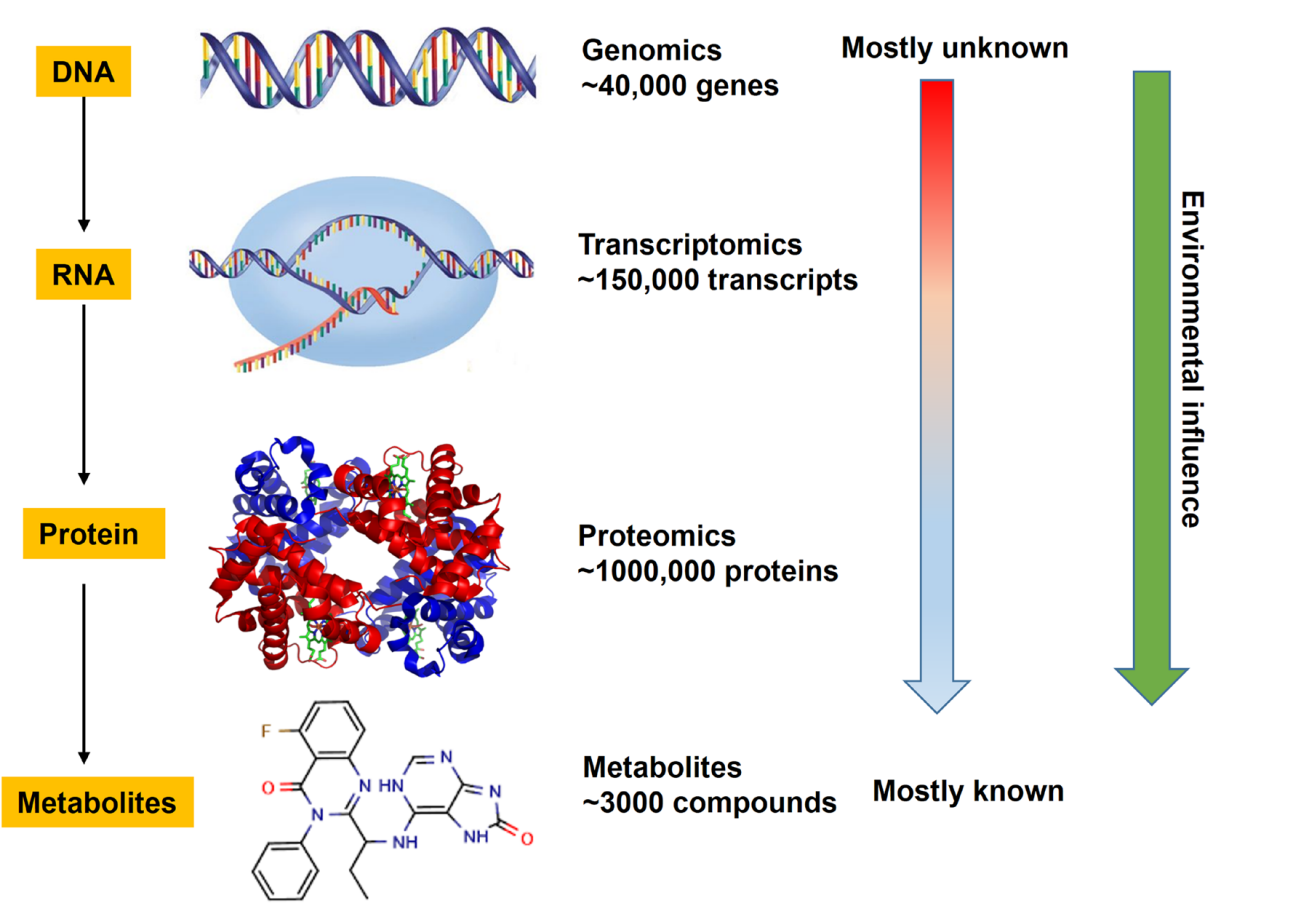
The advantages of metabolomics over other omics

Metabolism is a general term that is used to describe all biochemical reactions occurred in the body under the regulation of genes and proteins according to the central dogma of molecular biology. Metabolites refer to various small molecule compounds (molecular weight ≤1500 Da) involved in these biochemical reactions. Metabolites play an important role in maintaining the normal physiological function of human cells and organs, and they are the key components for intercellular signal transduction. A correct interpretation of this “language” is essential to understand pathological mechanisms of diseases and search early diagnostic markers. And the metabolomics is the right tool for interpreting these “languages”. Metabolomics is a new technology of systems biology developed in the post-genomic era, and it is the key technique in the China’s Middle- and Long-Term Plan for Development of Science and Technology (2006-2020). The purpose of metabolomics is to determine all small molecule metabolites in organisms. Compared to the genomics, transcriptomics and proteomics which can only tell us what may happen in organisms, the metabolomics can directly and accurately reflect the current status of organisms and tell us what has exactly happened in the organisms [2]. In contrast, transcriptomics and proteomics are very inadequate to monitor the cell function because there is no simple relationship between mRNA or protein level and metabolism due to RNA splicing or post-translation. Metabolome is much smaller than proteome and genome makes it relative simple for data analysis (Figure 1). There are only about 3000 commonly used metabolites in the key metabolic pathways, while >40,000 genes in the genome. Moreover, metabolome is the output of gene-environment interaction and reflects the environmental influence (Figure 1).

With the continuous development of testing instruments and equipment, the next-generation metabolomic technique based on the high performance liquid chromatography coupled with tandem mass spectrometry (LC-MS/MS) has significant improvement in both sensitivity and accuracy compared to the traditional metabolomic technique with single quadrupole mass dection. The next-generation metabolomics is becoming a new powerful tool for the cancer diagnosis and treatment. In this article, the development of next-generation metabolomics in recent years (2009-August, 2017) as well as the research progress in terms of screening of biomarkers for the early diagnosis of lung cancer, pathogenesis of lung cancer and guiding the precision medicine for lung cancer has been summarized.

### Next-generation metabolomic platforms

Traditional metabolomics started between 1998 and 2000, and the first article was published on Nature Biotechnology related to plant metabolomics [3]. In the traditional metabolomics, gas chromatography tandem mass spectrometry (GC-MS), nuclear magnetic resonance (NMR) and high performance liquid chromatography UV detector (LC-UV) were mainly used to screen the metabolites in the samples, and the main disadvantage of the traditional technology was the small metabolome coverage, and the number of metabolites which could be accurately determined was limited. The next-generation metabolomics has emerged after 2010, and it is mainly benefited from the development and application of the fast scanning tandem mass spectrometry (MS/MS) and high-resolution tandem time-of-flight mass spectrometry (TOF/TOF) as well as the large-scale metabolites identification database (e.g. HMDB & KEGG). Gary Siuzdak from The Scripps Research Institute (US), Oliver Fiehn from the University of California, Davis, Joshua Rabinowitz from the Princeton University and Jeremy Nicholson from the Britain Imperial College are all internationally renowned scholars in metabolomics.

**Figure 2:**
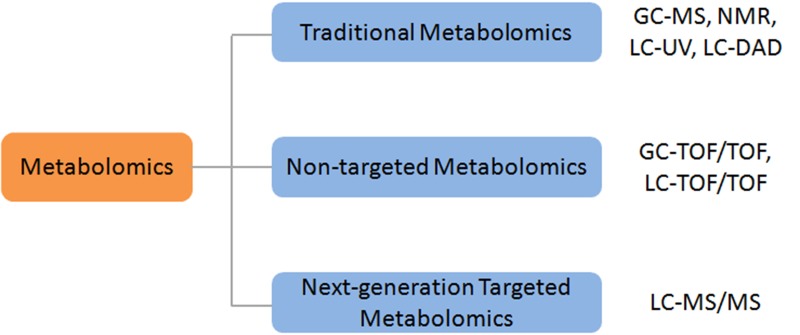
The classification of metabolomics and common instrument platform Abbreviations: gas chromatography(GC), mass spectrometry(MS), nuclear magnetic resonance(NMR), liquid chromatography(LC), diode-array detector(DAD), time-of-flight(TOF).

**Figure 3:**
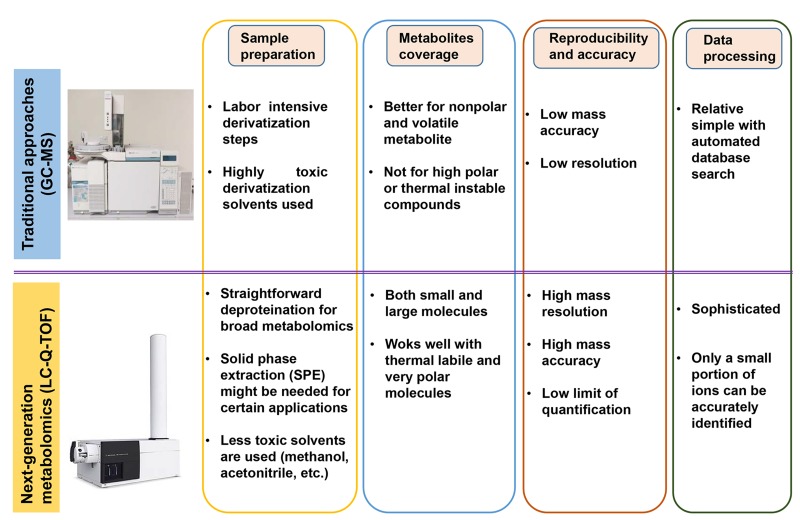
The comparison of next-generation metabolomics platforms with the traditional approaches

According to the different detection methods for the target metabolites, metabolomics can be divided into two categories: non-targeted metabolomics and targeted metabolomics. In the non-targeted metabolomics, all small molecular ions (50 Da∼1500 Da) in the test samples are fully scanned so that the ion mass spectrum and fragment ions mass spectrum are obtained. The metabolites are identified by comparing with database and standard substance verification. In the targeted metabolomics, an accurate determination of known metabolites is performed to understand how the metabolites would change under the condition of various diseases. The next-generation of non-targeted metabolomics and targeted metabolomics has its own advantages respectively and they are complementary to each other. In Figure 2, the classification of metabolomics and common instrument platforms are listed. The differences between the next-generation approaches and traditional metabolomic methods were summarized in Figure 3. Compared with the traditional approaches, the sample preparation procedures are straightforward. In most cases, to avoid any loss of metabolites during extraction, only a deproteination step using organic solvents is required for broad metabolomics. In certain applications, phospholipids might need to be removed to prevent the strong ion suppression. Traditional platforms had limited coverage of the metabolome. While, the next-generation metabolomics covers the majority of the metabolome and works well with both polar and nonpolar metabolites (Figure 3). In term of data processing, metabolomic results from next-generation platforms are more complicated than the traditional approaches which have a comprehensive mass spectrum database for assisting of compound identification. Even though thousands of ions and peaks are detected, only a small portion can be accurately identified.

The sample types for next-generation metabolomics can be any types of biological fluids and tissues (Table 1). The liquid samples usually need to be ≥ 50 μl and 20 mg for tissues. The general solvents are acetonitrile, methanol and isopropyl alcohol (IPA). Volatile modifiers are used including ammonium hydroxide, formic acid, ammonium acetate, ammonium carbonate and ammonium bicarbonate. There are several free data processing and statistical analysis tools for next-generation metabolomics data such as Metaboanalyst, Metabolite Set Enrichment Analysis (MSEA), Metlin, BioStatFlow and Human metabolome databse (HMDB).

**Table 1:** The setup of next-generation metabolomics platforms

Items	Next generation platforms
**Sample types**	Any biological fluids and tissues
**Sample size**	≥ 50 μl for liquid samples and 20 mg for tissues
**Equipment**	LC coupled with Quadruple, Ion trap, TOF, Qrbitrap
**Solvent needed**	Methanol, acetonitrile, IPA,
**Modifiers**	Formic acid, ammonium hydroxide, ammonium acetate, ammonium bicarbonate, ammonium carbonate
**Columns**	HILIC, reverse phase, normal phase
**Data analysis software**	Metaboanalyst; MSEA; Metlin; BioStatFlow; HMDB

Abbreviations: liquid chromatography(LC), time-of-flight(TOF), isopropyl alcohol(IPA), hydrophilic interaction chromatography(HILIC), metabolite set enrichment analysis(MSEA), human metabolome databse(HMDB).

### Applications of next-generation metabolomics in the lung cancer research and personalized medicine

**Figure 4:**
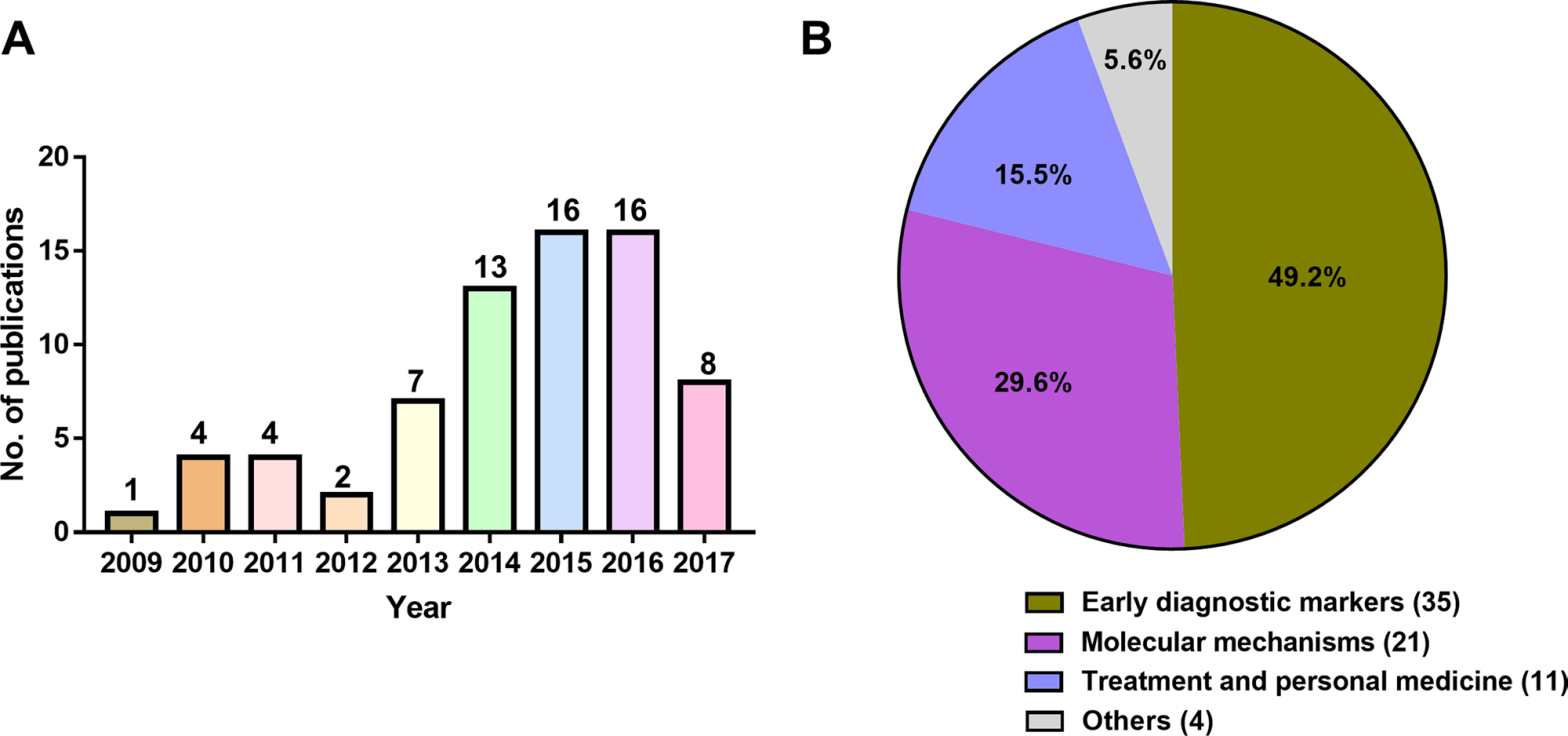
Articles on metabolomics of lung cancer published in the period of 2009- August, 2017 **(A)** Number of articles published in each year. **(B)** Content classification of the articles published. Data was obtained from PubMed using the key word of “lung cancer AND metabolomics” and “lung cancer AND metabolic profiling”.

The application of metabolomics in cancer research started about 10 years ago, and it has been gradually recognized by the researchers along with the research hotspot of cancer metabolism. In recent years, the research of metabolomics in cancers, especially in lung cancer cases has been progressing rapidly, and it is expected to become a new hotspot in the lung cancer research field. Retrieve articles in the PubMed using the retrieval terms of “lung cancer AND metabolomics” and “lung cancer AND metabolic profiling”, a total of 71 papers on the lung cancer metabolomics (review papers are not included) have been published in the period of 2009 - August, 2017 in which more than 50% of papers were published in recent 2 years (Figure 4A). Amongst the 71 articles, 35 (49.2%) articles are on the search of biomarkers for the early diagnosis of lung cancer, highlighting the potential role of next-generation metabolomics in lung cancer early diagnosis. Twenty-one articles are on the lung cancer pathogenesis research, and 11 articles are on the therapeutic effect of drug therapy for lung cancer and precision medicine regimens (Figure 4B).

### Next-generation metabolomics in the search of biomarkers for the early diagnosis of lung cancer

There are certain differences in the genomes of cancer cells and tissues in different cancer patients. Due to the heterogeneity of cancer cells, the accuracy and applicability of many diagnostic markers for genes and proteins are greatly reduced, and these markers for genes and proteins may be effective for some patients, while ineffective for others[4]. Metabolic biochemical reaction is an ancient, multi-species similar and conservative process that is strictly regulated by the organism, therefore, the difference between individuals is much smaller than that of genes and proteins. It can also be seen from the quantity of genes, proteins and metabolites that the complexity of metabolism is much lower than that of genes and proteins. In human genome, at present, there are about 20,000-25,000 known protein-coding DNA genes and in the proteome there are about 250,000-1,000,000 proteins, while in the human body there are only about 2000-2500 small molecule metabolites and these metabolites construct nearly 70 main metabolic pathways (Figure 1). Therefore, the metabolomics has great application potential for the search of reliable tumor metabolic markers and it is especially meaningful for cancers which are not easily to be diagnosed in the early stage, such as lung cancer.

The metabolic markers which could be potentially used for the lung cancer early diagnosis had been summarized in Table 2. There are two principles to be followed in terms of taking biological samples for early diagnosis: non-invasive and convenient. For the lung cancer, the main samples collected are serum, plasma and urine [37]. Since the lung is closely connected to the respiratory tract, samples of sputum, expiration concentrate and bronchoalveolar lavage fluid (BALF) are also collected in some studies. Currently the screened lung cancer diagnostic biomarkers are mainly divided into 4 categories: (1) Energy-related metabolites such as glucose and citric acid. Glucose provides energy for the growth of lung cancer cells by means of glycolysis and tricarboxylic acid cycle. The up-regulation of glycolysis and tricarboxylic acid cycle in the cancer cells is one of the known main pathways for cancer cell proliferation [38]. Glucose (mainly is D-glucose isomer) can activate the glucose-regulated proteins and then promote the lung cancer cell proliferation and invasion [39]. Acylcarnitines are involved in the fatty acid oxidation in mitochondria, which is the main source of cellular energy production [40]. Acylcarnitines are also the indicators of mitochondrial normal functions. Serum acylcarnitines were found to be significantly lower in early NSCLC [34]. (2) Amino acids and protein biosynthesis such as threonine, alanine and glutamic acid. These amino acids are important nitrogen sources for lung cancer cells. Besides, glutamic acid is also the carbon source for anaplerosis of tricarboxylic acid cycle. (3) Metabolites related to the cell membrane synthesis: such as choline, carnitine and fatty acids. Choline plays a quite important role in the lung cancer cell metabolism, and it’s the initial point of the synthesis of lipid materials and provides raw materials for cell membrane synthesis during the process of rapid cancer cell amplification. Choline provides also methyl for the DNA methylation, and this methylation process can interfere with the DNA repair and gene expression in normal cells [41]. Glycerol is the critical molecule in lipids biosynthesis and the backbone of all membrane glycerophospholipids. In a recent case study, glycerol was discovered to be dramatically increased in the serum of lung cancer patients [35]. Besides, lactic acid is also a potential metabolic marker for the early diagnosis of lung cancer. Lactic acid can reduce the extracellular pH and induce cancer cell metastasis. Lactic acid can induce immunosuppression in local tissues and then promote the proliferation of cancer cells [42, 43]. (4) Gut microbiome metabolites. Gut microbiome dysbiosis was recently found to be essential in the pathogenesis of lung cancer through gut-lung axis [44]. Upregulation of gut microbial metabolite benzaldehyde was shown to well differentiate the early cancer patients from healthy controls with 100% sensitivity and 95% specificity [36].

**Table 2:** Metabolic markers for the early diagnosis of lung cancer

Metabolite	Sample type	Function	References
**Glycerol, phosphoric acid**	Bronchoalveolar lavage fluid	Discriminate lung cancer patients and healthy controls	[5]
**Hydroxylamine, threonine**	Serum	Progression of lung cancer	[6]
**Monopalmitin, benzyl alcohol**	Expiration concentrate	Discriminate lung cancer patients and healthy controls	[7]
**Phosphatidyl ethanolamine**	Serum	Discriminate malignant and benign pulmonary nodules	[8]
**Putrescine, cysteamine**	Sputum	Discriminate lung cancer patients and healthy controls	[9]
**Glutamic acid, choline, threonine**	Serum	Discriminate non-small cell lung cancer patients and healthy controls	[10]
**Threonine, acetic acid, acetone**	Expiration concentrate	Differentiate lung cancer patients and healthy controls	[11]
**Lactic acid, inositol, valine**	Tissue	Discriminate malignant and benign pulmonary tissues	[12]
**Choline, carnitine**	Tissue	Discriminate lung cancer tissues and normal tissues	[13]
**Diethyl spermine**	Serum	Discriminate non-small cell lung cancer patients and healthy controls	[14]
**Glutamic acid, aspartic acid, xylose**	Serum and plasma	Discriminate lung adenocarcinoma patients and healthy controls	[15]
**Phosphorylcholine, sphingosine**	Serum	Discriminate lung cancer patients and healthy controls	[16]
**Glycerol, lactic acid, tryptophan**	Plasma	Discriminate non-small cell lung cancer patients and normal persons	[17]
**Benzoic acid, lactic acid, glucose**	Plasma	Discriminate lung cancer patients, smokers and non-smokers	[18]
**Glucose, cysteine, glucosamine**	Tissue	Discriminate malignant and benign pulmonary tissues	[19]
**Bilirubin**	Serum	Predict the lung cancer risk in smokers	[20]
**Octanedioic acid, tetrahexose**	Sweat	Discriminate lung cancer patients and normal persons	[21]
**Hydroxybutyrate, alanine**	Cerebrospinal fluid	Distinguish lung cancer leptomeningeal metastases	[22]
**Citric acid, choline, lysine**	Serum	Discriminate non-small cell lung cancer and chronic obstructive pulmonary disease	[23]
**Choline, linoleic acid**	Serum	Discriminate lung cancer patients and normal persons	[24]
**Carnitine, acetylcarnitine**	Urine	Discriminate non-small cell lung cancer patients and normal persons	[25]
**Creatine ribonic acid, acetylneuraminic acid**	Urine	Discriminate non-small cell lung cancer patients and normal controls	[26]
**Alanine, threonine, linoleic acid**	Plasma	Discriminate lung adenocarcinoma patients and normal persons	[27]
**Citric acid, choline, leucine**	Serum	Discriminate lung cancer and chronic respiratory disease	[23]
**Linoleic acid, hydroxy isobutyric acid**	Serum	Discriminate lung cancer patients and normal persons	[28]
**Glutamic acid, erythritol, palmitic acid**	Plasma	Discriminate small cell lung cancer patients and healthy controls	[29]
**Alanine, valine, isoleucine**	Plasma	Discriminate non-small cell lung cancer patients and normal persons	[30]
**Hippuric acid, hydroxy isovaleric acid, creatinine**	Urine	Discriminate lung cancer patients and healthy controls	[31]
**Tryptophan, hippuric acid, threonine**	Urine	Discriminate lung cancer patients and normal persons	[32]
**Taurine, hippuric acid, carnitine**	Urine	Discriminate lung cancer patients and normal persons	[33]
**Carnitine, propionylcarnitine, tyrosine, methionine, malic acid, histidine, 5-oxo-proline**	Serum	Discriminate non-small cell lung cancer patients and normal persons	[34]
**Glucose, tyrosine, glycerol, valine**	Serum	Discriminate lung cancer patients and non-cancer patients	[35]
**Benzaldehyde, urea, isoleucine, glycolic, phenylalanine**	Serum	Discriminate early stages of lung cancer patients and normal persons	[36]

### Next-generation metabolomics in the pathogenesis and classification of lung cancer

In the past 40 years since the discovery of gene and the birth of molecular biology, almost all of studies on chronic diseases, especially cancers are based on the premise that cancer is a genetic in origin disease. Especially after the development of gene sequencing, a large amount of resources have been devoted to the studies of whole genome sequencing, transcriptomics and single nucleotide polymorphism [45]. A number of important research achievements have been made, while compared to the huge investment, the cognitive output on lung cancer molecular mechanism is quite low [46]. Metabolic disorder has been brought into focus with the continuous development of metabolomic technique. In fact, metabolites are the final result of gene-environment interaction and are more sensitive than genes and proteins, and metabolites can reflect the influence of environment factors on organisms more effectively. It’s especially suitable for the research of diseases which are evidently influenced by environmental pollution and smoking, such as lung cancer.

Currently, certain progress has been made in exploring lung cancer cell metabolism by the next-generation metabolomics. The change in cell metabolism is a hallmark event of cancer incidence, and this acknowledge has become the consensus of academic community [47]. The main research results on lung cancer metabolism are summarized as follows:

#### Up-regulation and reprogramming of glycolysis and tricarboxylic acid cycle

**Figure 5:**
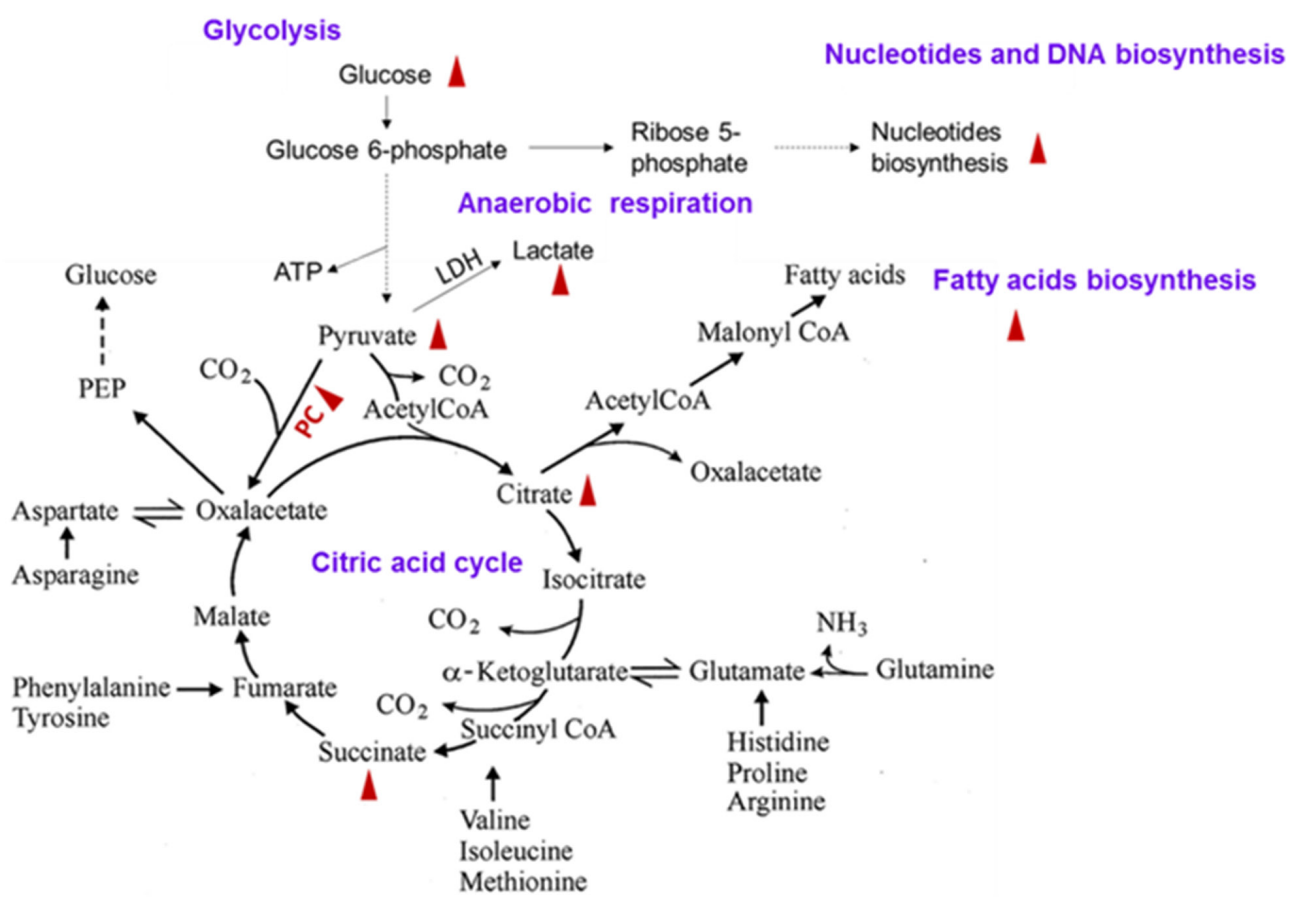
Up-regulation of tricarboxylic acid cycle, glycolysis, and fatty acid and nucleotide synthesis pathways in the lung cancer cells The red triangle means that the concentration of metabolite was significantly increased in the lung cancer tissues and cells. The dotted arrow represents intermediate multi-step reactions.

Uncontrollable cell proliferation is the common feature shared by lung cancer cells and other cancer cells. Compared to the normal cells, lung cancer cells need more energy to realize the rapid cell proliferation. Glycolysis and tricarboxylic acid cycle are the main pathways to generate ATP in the aerobic respiration of the human body. It was found by Teresa et al. using ^13^C isotope labelling metabolomics that the levels of lactic acid, glucose and citric acid in the lung cancer tissues are significantly increased compared to those in the normal tissues, which indicates an up-regulation of glycolysis and tricarboxylic acid cycle [48]. Whereafter, it was found by this research team in a study on NSCLC that the up-regulation of glycolysis and tricarboxylic acid cycle not only provides sufficient ATP energy for lung cancer cells, but also promotes the synthesis of fatty acid and nucleotide, so that the proliferation of lung cancer cells is ensured (Figure 5). Pyruvic carboxylase is the key metabolic enzyme to up-regulate the two pathways, and it plays an important role in the early phase of NSCLC proliferation [49].

#### Up-regulation of phospholipid metabolic pathways and fatty synthesis

Phospholipids are important constituent parts of cell membrane, accounting for more than 40% of the cell membrane composition. Besides, after the lipid is synthesized in the cell, it’s released into the blood in the form of exosome and combines with lipoprotein forming cell signaling factor, and then the cell signaling factor binds to the cell membrane, so that the cell proliferation and apoptosis are regulated. Rocha et al. determined the concentrations of metabolites in the lung cancer tissues by NMR-based metabolomics and then compared the concentrations to those in the normal tissues, and it was found that the contents of phosphoryl choline and glyceryl phosphoryl choline in the lung cancer tissues were significantly increased, which indicates an up-regulation of phospholipid metabolic pathways [50]. Whereafter, Chen et al. conducted metabolomics analysis with respect to different types of lung cancer tissues (squamous-cell carcinoma, adenocarcinoma, small cell lung cancer and malignant mesothelioma) using the same NMR-based technology and the up-regulation of phospholipids metabolic pathways in lung cancer cells was further confirmed [51].

#### Metabolic differences between different types of lung cancer

Metabolomics has great potential in differentiating different types of lung cancers. Study results have demonstrated that for different types of lung cancers, the metabolism processes differ significantly. For example, lung adenocarcinoma and squamous cell lung carcinoma are two main subtypes of non-small cell lung cancer. Rezola et al. compared the cellular metabolisms of these two subtypes lung cancer with each other using metabolomics, and it was found that there were significant differences in the metabolites such as glycerinum, acetone and acetoacetic acid between the two subtypes [52]. KRAS is a common carcinogenic mutant gene in the non-small cell lung cancer. KRAS mutation will activate the PI3Ks signal transduction pathway, and then activate the cancer cell proliferation and tumor growth. Using the next-generation targeted metabolomics method, Caiola et al. explored the metabolic differences between the lung cancer cells with KRAS mutation and the wild type non-mutated lung cancer cells, and it was found that the level of glutamine was significantly decreased in the lung cancer cells with KRAS mutation, and this finding can be used to distinguish this type of mutation [53].

### Next-generation metabolomics in the lung cancer precision medicine

Precision medicine is a new concept that has arised in recent 2 years with the development of new technologies of systems biology, such as genomics, transcriptomics, epigenetics, proteomics and metabolomics. In 2015, Precision Medicine Initiative was initiated in USA. The key specific projects of Ministry of Science and Technology of China for 2016 started the precision medicine scientific research activities in China. In the precision medicine, the influences of environment, genetics and lifestyles on individuals are sufficiently considered. The main objective of precision medicine is to realize a personalized analysis for individual patient using new technologies of systems biology and precisely reveal the cause of diseases and the therapeutic target so as to find out the optimal therapeutic regimen. Currently the precision medicine is still in the scientific research stage, and the application of precision medicine in cancers is the field closest to clinical application. The non-small cell lung cancer has become a typical case for precision medicine [54]. As an effective supplement to the new technologies of systems biology and gene sequencing technology, the next-generation metabolomics plays an important role in precision medicine [55].

**Figure 6:**
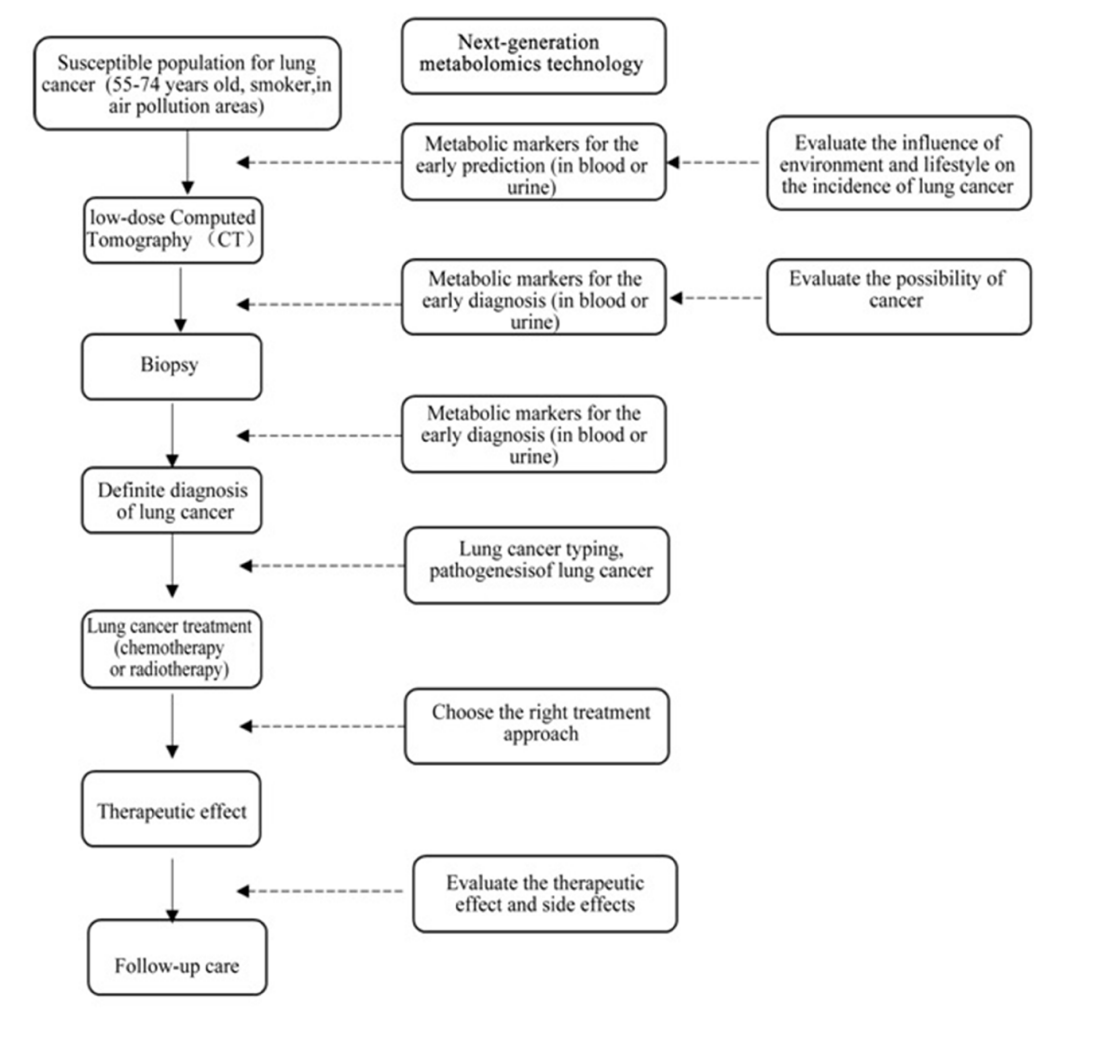
Application of next-generation metabolomics in the lung cancer precision medicine

Robles et al. summarized the roles of integrating the next-generation metabolomics and other approaches in lung cancer precision medicine [56]. As demonstrated in Figure 6, the metabolomics can identify the metabolic markers for lung cancer early prediction and diagnosis, reveal the pathogenesis of individual lung cancer case and distinguish different types of lung cancers; besides, another important role of the metabolomics is to evaluate the effectiveness of drugs and therapeutic approaches on individual lung cancer patients. Individual lung cancer patient reacts differently to the radiotherapy or chemotherapy. These differences are closely bonded to the body weight, age, diet and lifestyle of patients and the difference in metabolism is the overall performance of these differences *in vivo*. Cis-platinum is a common chemotherapeutics for lung cancer, but it can induce renal toxicity in many patients. Teng et al. evaluated the protective effect of Chinese traditional medicine Qiongyugao against the renal toxicity in patients treated with cis-platinum using the metabolomics. They found that Qiongyugao could reduce the level of urea and creatinine in the plasma of patients treated with cis-platinum so that the renal toxicity induced by the use of cis-platinum was reduced [57].

The next-generation metabolomics can also play a role in the precise medicine in the surgical treatment of lung cancer. It’s reported that by the high-resolution magic angle spinning magnetic resonance spectroscopy and metabolomics analysis, the boundaries of breast cancer tissues can be determined and the breast cancer tissues can be differentiated from the adjacent normal tissues, guiding the precision resection of breast tumors [58]. This idea can also be used in the surgical treatment of lung cancer to guide the precision resection of lung lobe in patients with lung cancer.

### Issues and future directions

Metabolomics is a relatively young branch of academic field compared to the genomics, transcriptomics and proteomics, and its application in the lung cancer has just started. Currently there are several difficulties in the lung cancer research using metabolomics technology: (1) For the screened lung cancer markers, effective clinical validations and comparison among various diseases are lacked. At present, some lung cancer metabolic markers have been screened, but they are mainly screened from a limited number of cases, and the following large-scale clinical validations are absent and it is still at the scientific research stage. Most of the lung cancer markers are screened by the comparison of metabolites in the lung cancer patients to those in the normal people and validations are still missing for the differentiation of lung cancer associated with other diseases. (2) For most metabolic markers, the related studies were focused on the qualitative comparison and quantitative analysis is lacked, and the testing of absolute concentrations of the metabolic markers in tissues, cells, blood and urine are lacked, which largely limits the clinical application of these markers. (3) In terms of lung cancer mechanism, software tools and platforms integrated of multiple technologies such as genomics, proteomics, metabolomics and imageology are still not available. (4) In the precision medicine field, a big data integration and collection platform is still lacked. The NCI-MATCH (Molecular Analysis for Therapy Choice Trial) program launched by the National Cancer Institute (US) in May 2016 initiated the largest and most rigorous precision tumor research in human history, of which the precision medicine research on NSCLC is one of the most important parts and it has been separated as ALCHEMIST (The Adjuvant Lung Cancer Enrichment Marker Identification and Sequencing Trial) clinical trial program [59].

In summary, as an interdisciplinary subject, the metabolomics is integrated of many related disciplines including analytical chemistry, molecular biology, biochemistry, bioinformatics and computer big data science. The metabolomics will play more and more important roles in the early diagnosis of lung cancer, molecular mechanism research and precision medicine.
